# The foundation of precision medicine: integration of electronic health records with genomics through basic, clinical, and translational research

**DOI:** 10.3389/fgene.2015.00104

**Published:** 2015-03-17

**Authors:** Marylyn D. Ritchie, Mariza de Andrade, Helena Kuivaniemi

**Affiliations:** ^1^Biochemistry and Molecular Biology, Center for Systems Genomics, The Pennsylvania State UniversityUniversity Park, PA, USA; ^2^Institute of Biomedical and Translational Informatics, Geisinger Health SystemDanville, PA, USA; ^3^Division of Biomedical Statistics and Informatics, Department of Health Science Research, Mayo ClinicRochester, MN, USA; ^4^The Sigfried and Janet Weis Center for Research, Geisinger Health SystemDanville, PA, USA; ^5^Department of Surgery, Temple University School of MedicinePhiladelphia, PA, USA

**Keywords:** electronic health records, precision medicine, genomic medicine, EHR, genomics

The members of the Genomics Workgroup in the Electronic Medical Records and Genomics (eMERGE) network (Gottesman et al., 2013) led the development of a Special Topic in Frontiers in Genetics titled “Genetics Research in Electronic Health Records Linked to DNA Biobanks^1^.” The goal was to publish papers representing the diverse research ongoing in the integration of electronic health records (EHR) with genomics through basic, clinical, and translational research. The special topic with its 18 papers is extremely timely given the recent announcement of the Precision Medicine initiative by the White House^2^, which includes the potential to build a biobank of 1 million Americans with rich, phenotypic data—likely from EHR. eMERGE has, therefore, served as an excellent test case for how a 1 million person project might work across several medical centers, EHR systems, and genetic datasets.

The first group of papers (Almoguera et al., 2014; Crawford et al., 2014; Crosslin et al., 2014; Verma et al., 2014) belonging to this special issue presents the eMERGE network and its contribution to genomics. The paper by Crawford et al. (2014) describes the initial goal of eMERGE network that was to explore the utility of EHRs in genomics and whether the phenotypes identified through algorithms using EHRs combined with the genome-wide genotypes could lead to fruitful results. The beginning of the network included individual genotype datasets that were later combined to form the merged eMERGE datasets and the combination with phenotypes from EHRs has led to new genomic discoveries. All of these steps subsequently lead to new goals that have included next generation sequencing and clinical practice. The second paper (Verma et al., 2014) introduces the new challenges involved in merging genotype data from different eMERGE sites. Since genotypes at different sites were derived from different genotyping platforms it was impossible to create a single merged data file based on raw genotype data alone. The solution was first to impute each site separately using the same software and pipeline, and then merge the imputed genotype data sets to form a combined dataset. The authors used two different imputation software packages and describe the challenges involved in using diverse ethnic populations and different genotype platforms, which lead to a complete pipeline that not only performs imputation but also ensures appropriate quality control for merging genotype data sets. The final eMERGE imputed data set is a valuable resource for genomic discovery by using the clinical data generated by the EHRs and will be available in dbGaP soon. The third paper (Crosslin et al., 2014) discusses the issues of population stratification and genotype platform bias. Principal components analysis (PCA) is commonly used to control for population stratification; however other factors such as local genomic variation, multiple study sites and multiple genotyping platforms may also increase the correlation patterns in the PCA. In this paper Crosslin et al. (2014) provided an alternative approach to PCA by deriving components from subject loadings determined by the 1000 Genomes reference sample that avoid the bias introduced by site and genotype platform effects. This alternative approach was applied successfully in the eMERGE genome-wide association study (GWAS) for venous thromboembolism in African Americans. The fourth paper in this group by Almoguera et al. (2014) evaluated the utility of large imputed genotype data sets to identify subjects with *TPMT* defective alleles. They used around 87,000 samples from the biobank at the Children's Hospital of Philadelphia. For 12 samples also Sanger sequencing data were available allowing comparison between the imputed and observed genotypes. The concordance rate between the non-carriers of the risk alleles was 98.88%; however the sensitivity of imputation for homozygous carriers was ~80%. The authors recommend using imputation of *TMPT* alleles as a first step to screen individuals at risk.

The papers of group 2 (Kullo et al., 2014a; Mitchell et al., 2014; Namjou et al., 2013; Parihar et al., 2014; Ye et al., 2014) describe different applications of the EHR derived phenotypes. The first paper (Namjou et al., 2013) investigated whether the common variants in the genes *FTO*, *MC4R* and *TMEM18* associated with BMI in adults are also associated in pediatric population in the eMERGE network. First they used a linear regression model with the dependent variable BMI, adjusted for age, sex, and PC by cohort; and then meta-analyzed the results using a weighted z-score approach. They not only reproduced the findings for the pediatric cohorts but also identified a novel locus at *COL6A5*. The second paper (Mitchell et al., 2014) described the issues when using cases generated from Stroke Genetics Network (SiGN) and using genotyped controls from eMERGE leading to recommendations regarding the controls selection, population stratification, imputation, and association analysis. The third paper by Kullo et al. (2014a) performed a two-stage association study to identify variants associated with peripheral arterial disease. The first stage was a GWAS adjusted for age and sex in subjects of European ancestry. In the second stage the top 48 SNPs were replicated in new set of cases and controls. One single nucleotide polymorphism (SNP) in the *ATXN2-SH2B3* gene was significant where this SNP is in high LD with a missense variant in *SH2B3*, a gene that is related to immune and inflammatory response pathways and vascular homeostasis, indicating a pleiotropic effect. The fourth paper (Parihar et al., 2014) carried out a GWAS for lipid-related phenotypes derived from the EHR using the Metabochip array. These phenotypes consist of laboratory, anthropomorphic and demographic data on a cohort of extremely obese subjects. They replicated 12 of 21 previously identified lipid-associated SNPs demonstrating the validity of using phenotype data available from the EHR and the usefulness of the Metabochip array. The fifth paper (Ye et al., 2014) performed GWAS to identify genetic variants associated with diseases caused by *Staphylococcus aureus* infection. They used different approaches to identify the genetic susceptibility from single SNP, gene set and pathway. No SNPs or genes were found to be genome-wide significant leaving with the speculation that multiple genes contribute to the severity of the infection.

The third group of papers (Connolly et al., 2014; Cronin et al., 2014; Namjou et al., 2014; Patel et al., 2014; Sun et al., 2014) in this special issue focused on more complex analyses of the genome including copy number variants (CNV), pleiotropy combined with phenome-wide association studies (PheWAS), and epistasis (gene-gene interactions). The first paper by Namjou et al. (2014) describes the first PheWAS in a pediatric cohort based on 4268 samples and 2476 sSNPs selected from previously published GWAS studies. A total of 539 EMR-derived phenotypes were explored. The authors identified a number of known associations which serve as a positive control as well as several novel associations including *NDFIP1* associated with mental retardation and *PLCL1* associated with developmental delays and speech disorder. The second paper by Cronin et al. (2014) is another PheWAS, focused on one specific gene, *FTO*, in 10,487 individuals from the eMERGE network and another 13,711 individuals from the Vanderbilt biobank BioVU. They identified highly significant associations between *FTO* and obesity, type II diabetes, and sleep apnea, all of which are expected for variants in this gene. A novel association was identified between *FTO* and fibrocystic breast disease. The third paper by Sun et al. (2014) is a review of methods to filter genome-wide SNP data to explore epistasis models effectively. There are a number of challenges with the search for epistasis in genome-wide data including the computational complexity of exploring that many different combinations of variables which can exceed computational feasibility as well as the magnitude of the multiple testing incurred by testing the genome in exhaustive interaction analyses. The authors discuss two different filtering approaches, namely using statistical effects or biological prior knowledge. Strengths and weakness of these different strategies are described as well as additional resources for consideration before a genome-wide epistasis analysis is initiated. The fourth paper by Connolly et al. (2014) is a review on recent research in the area of CNV including successful applications in rare and common diseases. Methods for identifying CNVs from array-based genotyping data and sequencing data are described. Finally, how CNVs might be evaluated and used with medical records is discussed. The fifth paper of this group is by Patel et al. (2014) and describes quality control processes for whole exome sequencing data, specifically using Mendelian errors as a filtering strategy to minimize errors. The group developed the Cincinnati Analytical Suite for Sequencing Informatics (CASSI) to store sequencing files, metadata, and others. Their data cleaning process can be used to improve the signal-to-noise ratio and improve the identification of candidate disease causative variants.

The fourth group of papers (Goldstein et al., 2014; Kullo et al., 2014b; Schrodi et al., 2014; Sleiman et al., 2014) belonging to this special issue discusses the use of genetic data together with EHR-derived clinical data in clinical settings. The first one of these papers (Sleiman et al., 2014) used imputed GWAS data to study two loss-of-function variants in the *PCSK9* gene. The study of 8028 genotyped biobank participants with extensive laboratory data from the EHR demonstrated that EHR-linked biobanks are a rich resource for exploring functional aspects of genetic variants. The second paper (Schrodi et al., 2014) is a review article about genetic-based prediction by Schrodi et al. (2014) and it provides a comprehensive discussion about disease prediction using both genetic and clinical data, again highlighting the usefulness of available EHR-linked genetic data on large cohorts. As the title of their article reveals, predicting who is at risk for a given disease has turned out to be a difficult task. Currently the most promising results can be found in cancer genomics, population screening of rare Mendelian diseases, and pharmacogenetics. Developing prediction models for common complex diseases such as type 2 diabetes mellitus, stroke and inflammatory arthritis has been more challenging and the results have been disappointing. This was also evident in the third paper of this group (Goldstein et al., 2014) in which coronary heart disease was investigated in the NIH-funded Atherosclerosis Risk in Communities (ARIC) cohort. The authors combined a genetic risk score derived from 45 SNPs with a clinical risk score, but received only minimal improvement in discrimination and calibration statistics of the risk score. Schrodi et al. (2014) conclude their review article with a positive note pointing out that in the near future we can rely on having access to additional genome-wide data which might help in refining the risk prediction. These data will include whole genome and whole exome sequence data, and other omics data such as information on DNA methylation, histone modification, and the transcriptomes of different tissues. Additional advances leading to more refined phenotyping, and development of new, more robust computational approaches will contribute to improved accuracy in risk estimates. The last paper in the fourth group (Kullo et al., 2014b) deals with the key questions about returning results to patients and providers. The authors are from the eMERGE network and point out that one of the mandates of the network is to come up with the best practices for implementing genomic medicine. The goal is to have the clinically relevant genetic results in the EHR so that they are easily available for the practicing physician to be used at point-of-care. These results could be individual risk genotypes or combined risk scores. Each of the eMERGE network sites is carrying out a feasibility projects, e.g., the group at Icahn School of Medicine at Mount Sinai is using *APOL1* variants in African Americans to predict chronic kidney disease and investigators at Vanderbilt University have chosen 14 actionable pharmacogenetic variants to be returned to the EHR.

Precision medicine (See Footnote 2) is an important focus for biomedical, clinical and translational informatics in the current era. The manuscripts presented in this special topic are well positioned to educate and demonstrate the potential study designs, methods, strategies, and applications where this type of research can be performed successfully. The ultimate goal is to improve diagnostics and provide better, more targeted care to the patient.

## Conflict of interest statement

The authors declare that the research was conducted in the absence of any commercial or financial relationships that could be construed as a potential conflict of interest.

## Footnotes

^1^Available online at: http://journal.frontiersin.org/ResearchTopic/2198

^2^Available online at: WH.GOV/PRECISION-MEDICINE
